# Editorial: Precision oncology: integrating molecular mechanisms, organoid models, and omics technologies for personalized cancer care

**DOI:** 10.3389/fonc.2026.1898081

**Published:** 2026-06-22

**Authors:** Muharrem Okan Cakir, Betul Karademir-Yilmaz, G. Hossein Ashrafi, Mustafa Özdoğan

**Affiliations:** 1 School of Life Sciences, Pharmacy and Chemistry, Kingston University London, London, United Kingdom; 2 Department of Biochemistry, School of Medicine, Marmara University, Istanbul, Türkiye; 3 Department of Biochemistry, School of Medicine, Recep Tayyip Erdogan University, Rize, Türkiye; 4 Division of Medical Oncology, Antalya Memorial Hospital Cancer Centre, Antalya, Türkiye

**Keywords:** multi omics, organoid models, personalized oncology, precision oncology, translational oncology

Precision oncology has entered a new phase. The field is no longer defined only by the identification of a targetable mutation or the selection of a matched therapy. Instead, it increasingly depends on the integration of molecular mechanisms, multi-omics technologies, functional disease models, tumour microenvironmental context, hereditary risk assessment, and clinically realistic trial design. This Research Topic brings together these interconnected dimensions and illustrates how personalised cancer care is evolving from a genomics-centred concept into a broader translational framework.

A recurring theme across the collection is that cancer should be understood as a dynamic biological system rather than a static genetic disease. Tumour progression, metastatic dissemination, therapeutic resistance, and immune escape are shaped by interacting layers of regulation, including genetic alterations, epigenetic plasticity, post-translational modification, mitochondrial function, and microenvironmental adaptation. In this context, Turna and Demokan provide an important synthesis of epigenetic alterations in cancer metastasis, highlighting how DNA methylation, histone modifications, chromatin remodelling, and non-coding RNAs regulate epithelial–mesenchymal transition, metastatic plasticity, dormancy, and colonisation. Complementing this mechanistic perspective, Zhang et al. explore SUMOylation as a regulatory process involved in malignant progression and chemotherapy response, with SENP5 emerging as a potentially more selective therapeutic vulnerability than global SUMO pathway inhibition.

The Research Topic also demonstrates the growing importance of omics-driven classification and biomarker discovery. Huang et al. investigate mitochondrial permeability transition-driven necrosis-related genes in colorectal cancer and propose a prognostic model that connects cell death pathways, immune checkpoint expression, and predicted therapeutic sensitivity. Laaribi et al. apply next-generation sequencing to hepatocellular carcinoma in a Tunisian cohort, identifying germline and somatic alterations that may inform future population-specific molecular stratification. Similarly, Bahsi et al. describe multilocus inherited neoplasia allele syndrome in a Turkish cohort, emphasising that expanded germline testing can reveal complex inherited cancer risk patterns that do not always fit traditional single-gene hereditary cancer models.

These studies collectively show that precision oncology must account for both common and rare molecular contexts. In non-small cell lung cancer, Hu et al. examine uncommon EGFR p.L747P and p.L747S mutations and highlight the clinical challenge of interpreting rare variants when evidence is limited. Their findings reinforce the need for careful integration of molecular data, clinical response patterns, and published case-level evidence. In extensive-stage small cell lung cancer, Yang et al. review the transition from conventional chemoimmunotherapy toward molecular subtyping, DLL3-targeted agents, bispecific antibodies, antibody-drug conjugates, and cellular therapies. Together, these contributions reflect the widening therapeutic landscape of precision oncology, while also acknowledging the limits of current biomarkers and the need for more refined patient selection.

Functional modelling represents another essential pillar of this collection. The review by Yadav et al. discusses preclinical screening models in anticancer drug development, underlining the translational limitations of conventional two-dimensional systems and animal models while emphasising the promise of three-dimensional cultures, patient-derived organoids, organ-on-chip platforms, and computational approaches. Importantly, the clinical relevance of functional modelling is illustrated by Zhang et al., who report stable disease in metastatic castration-resistant prostate cancer following treatment informed by organoid drug screening. Similarly, Wert et al. present a case of relapsed osteosarcoma in which molecularly guided therapy, transcriptomic interpretation, *in vitro* drug testing, and tumour board recommendations contributed to an exceptional long-term response. These examples support the emerging view that molecular profiling is most powerful when paired with functional evidence of therapeutic vulnerability.

Precision oncology also requires more accurate diagnostic and clinical decision-making tools. Zhao et al. develop an integrated diagnostic panel for pleural effusion by combining methylation biomarkers, tumour markers, DNA ploidy analysis, and cytology. This multimodal approach reflects a broader movement toward diagnostic systems that reduce uncertainty by combining molecular, morphological, and clinical information. At the same time, precision medicine must confront structural limitations in evidence generation. Shi et al. show that multiple primary lung cancer remains markedly underrepresented in clinical trials, despite its increasing clinical recognition. Their analysis reminds us that precision oncology cannot be fully realised if complex patient groups are systematically excluded from research frameworks.

Taken together, the articles in this Research Topic present precision oncology as a translational continuum. Mechanistic insights help explain tumour behaviour; omics technologies identify molecular subgroups and vulnerabilities; organoid and functional models test therapeutic sensitivity; integrated diagnostics support clinical interpretation; and inclusive trial designs determine whether innovation reaches real-world patients. The collection therefore moves beyond a narrow biomarker-based view of precision medicine and supports a more comprehensive model in which treatment decisions are informed by biological complexity, functional validation, and clinical context.

The future of personalised cancer care will depend on this integration. Genomic alterations must be interpreted alongside epigenetic states, immune regulation, mitochondrial function, tumour heterogeneity, drug response modelling, hereditary predisposition, and patient-specific clinical realities. By bringing together diverse tumour types, methodologies, and translational perspectives, this Research Topic contributes to a more mature and clinically meaningful vision of precision oncology: one in which personalised treatment is not guided by isolated molecular findings alone, but by a multidimensional understanding of cancer biology in each patient.

